# Long-Term Outcomes of Patients with Pre-Existing Essential Tremor After SARS-CoV-2 Infection

**DOI:** 10.3390/diagnostics14242774

**Published:** 2024-12-10

**Authors:** Rachel Pakan, Roham Hadidchi, Yousef Al-Ani, Hannah Piskun, Katie S. Duong, Sonya Henry, Stephen Wang, Carine W. Maurer, Tim Q. Duong

**Affiliations:** 1 Department of Radiology, Albert Einstein College of Medicine and Montefiore Medical Center, Bronx, NY 10461, USA; rachel.pakan@einsteinmed.edu (R.P.); roham.hadidchi@einsteinmed.edu (R.H.); yousef.al-ani@einsteinmed.edu (Y.A.-A.); hrpiskun@gmail.com (H.P.); 2007katieduong@gmail.com (K.S.D.); sonya.henry@einsteinmed.edu (S.H.); shwang3@bidmc.harvard.edu (S.W.); 2 Department of Neurology, Renaissance School of Medicine, Stony Brook University, Stony Brook, NY 11794, USA; carine.maurer@stonybrookmedicine.edu

**Keywords:** long COVID, essential tremor, PASC, neurodegenerative diseases

## Abstract

Background/Objectives: Although COVID-19 has been linked to worse outcomes in patients with neurological disorders, its impact on those with essential tremor (ET) remains unclear. To investigate clinical outcomes of ET patients with and without COVID-19 three and a half years post-pandemic. Methods: 1074 ET patients were evaluated in this retrospective study in the Montefiore Health System from January 2016 to July 2023. Comparisons between ET patients with and without a positive SARS-CoV-2 polymerase chain reaction test were made. Outcomes included post-index date major adverse cardiovascular events (MACEs), new-onset sleep disturbances, fatigue, dyspnea, first-time fall, new-onset anxiety, new-onset depression, headache, new-onset imbalance, new-onset mild cognitive impairment, and all-cause mortality, adjusted hazard ratios (aHR) adjusting for covariates were calculated. Results: ET patients with COVID-19 had higher prevalence of pre-existing type-2 diabetes, depression, and anxiety compared to ET patients without COVID-19. COVID-19 was significantly associated with higher risk of MACEs, (aHR = 2.39 [1.49, 3.82]), new-onset sleep disturbance, (aHR = 2.12 [1.44, 3.13]), fatigue, (aHR = 1.83 [1.27, 2.65]), dyspnea, (aHR = 1.98 [1.40, 2.80]), first-time fall, (aHR = 4.76 [2.24, 10.14]), new-onset anxiety, (aHR = 3.66 [2.02, 6.64]), and new-onset depression, (aHR = 2.38 [1.20, 4.70]). COVID-19 was not associated with all-cause mortality. Conclusions: In patients with ET, COVID-19 significantly increases the risk of several long-term adverse health outcomes, but not mortality.

## 1. Introduction

Since the start of the COVID-19 pandemic, the SARS-CoV-2 virus has been associated with worsening of a wide array of chronic sequelae. Acute COVID-19 can trigger acute cardiovascular and pulmonary stress, a hypercoagulable state, and pathologic chronic hyperinflammation, which can manifest as chronic symptoms in some individuals. COVID-19 survivors have reported persistent symptoms [1,2,3,4,5,6,7,8], developed new disorders [9,10,11], or have had exacerbation or worsening of pre-existing conditions [12] months after acute COVID-19 had resolved [13,14]. These symptoms and disorders are commonly called long COVID.

Essential tremor (ET) is one of the common movement disorders, impacting approximately 1% of the population. It involves a 4–12 Hz involuntary action tremor of the bilateral upper extremities. While there are multiple proposed etiologies for ET, studies have identified distinct structural changes consistent with neuronal loss in the cerebellum, implicating neurodegenerative changes in the underlying pathogenesis of ET [15,16].

It is possible that in some with underlying neurological disease, COVID-19 could accelerate neurodegenerative progression, making these patients more susceptible to adverse clinical outcomes compared to non-COVID-19 individuals [17]. Previous cohort studies have shown the acute and longer negative impacts of COVID-19 on disease progression in a number of neurological disorders, including multiple sclerosis (MS) [18,19,20,21,22,23,24,25,26,27,28] and Parkinson’s disease (PD) [29,30,31,32]. Analyses investigating the effects of COVID-19 on patients with pre-existing ET have been limited to two case studies in Italy, in which both patients experienced acute worsening and significant disease progression at one-year follow-up [33,34]. No cohort studies thus far have explored the interplay between COVID-19 and pre-existing ET.

To investigate the long-term prognosis of ET patients with and without COVID-19 three and a half years post-pandemic, several post-infection endpoints were analyzed: all-cause mortality, major cardiovascular events (MACEs), as well as new-onset sleep disturbances, fatigue, dyspnea, first-time fall, new-onset anxiety, new-onset depression, headache, new-onset imbalance, and new-onset mild cognitive impairment (MCI). Data originated from the Montefiore Health System in the Bronx, which serves a diverse urban population, one of the epicenters of the early COVID-19 pandemic and subsequent surges of infection. We hypothesized that COVID-19 exposure is associated with worse long-term outcomes in individuals with ET.

## 2. Methods

### 2.1. Data Sources

The Einstein-Montefiore Institutional Review Board (#2021-13658) approved this retrospective single-center study with an exemption for informed consent. Deidentified health data from the Montefiore Health System’s electronic health records were extracted as previously described from 1 January 2016 to 1 July 2023 [9,12,35,36,37,38,39,40,41,42,43,44,45,46].

### 2.2. Study Cohort

One thousand and seventy-four individuals with underlying ET at index date were identified. For COVID-19 patients, the index date was the date of the first occurrence of a positive result from a SARS-CoV-2 polymerase chain reaction (PCR) test. For non-COVID patients who served as contemporary controls, index date was the first clinical visit at the health system after 1 March 2020. For inclusion, individuals in both groups had to have at least one return visit at least 14 days after index date. The last follow-up date was 1 July 2023.

### 2.3. Variables

Sex, race, ethnicity, and age at index date were the demographic variables. Presence or absence of the following comorbidities at index date was captured: type-2 diabetes (T2D), chronic obstructive pulmonary disease (COPD), chronic kidney disease (CKD), asthma, hypertension (HTN), chronic heart failure (CHF), and coronary arterial disease (CAD). Obesity included an obesity diagnosis from a clinician or a body mass index of at least 30. Tobacco use was obtained via self-reporting. For ET medications, first-line (propranolol, atenolol, or primidone) and second-line (clonazepam, lorazepam, diazepam, gabapentin, or topiramate) prescriptions were collected. To capture the severity of the acute phase of COVID-19, acute treatment data were collected: prescription of corticosteroids (prednisone, dexamethasone, hydrocortisone, and methylprednisolone) and antivirals (ritonavir and remdesivir), hospitalization during the acute phase of COVID-19, and intensive care unit or invasive mechanical ventilation (collectively referred to as critical illness).

### 2.4. Outcomes

The primary outcomes analyzed were the following: all-cause mortality at least 14 days after index date; MACEs (composite of new-onset heart failure, myocardial infarction, stroke, and cardiogenic shock) at least 30 days after index date. Secondary outcomes were defined as the first incidence of the following 30 days or more after index date: (i) new-onset of sleep disturbances (irregular sleep-wake pattern, central sleep apnea, or new-onset idiopathic insomnia), (ii) fatigue (patient-reported symptom that was recorded by a clinician), (iii) dyspnea, (iv) first-time fall, (v) new-onset anxiety (GAD-7 score of at least 10, GAD-2 score of at least 3, or generalized anxiety disorder diagnosis, GAD), (vi) new-onset depression (PHQ-9 score of at least 10 or major depressive disorder diagnosis), (vii) headache (idiopathic migraine or other headache), (viii) new-onset imbalance (non-congenital, non-genetic, and non-cerebrovascular accident-induced ataxia or incoordination), and (ix) new-onset MCI. Patients with a pre-index date history of sleep disturbances, falls, anxiety, depression, migraines, imbalance, and MCI or dementia were not included in the analysis of the respective outcome. OMOP Concept IDs for these outcomes are shown in Supplemental Table S1.

### 2.5. Analysis and Statistics

For data processing and analysis, the lifelines package was used in Python version 3.10.12, and the following RStudio version 4.3.2 (RStudio, PBC, Boston, MA, USA) packages were utilized: survival, survminer, and cmprsk, alongside GraphPad Prism 9 version 10.1.1 (GraphPad Software, Boston, MA, USA). For comparison of variables across groups, the chi-square test was used for categorical variables and the independent *t*-test for continuous variables. Survival analysis for all-cause mortality was performed using Kaplan–Meier curves and the log-rank test. Cumulative incidence curves and univariate Fine–Gray hazards analysis were used to account for death as a competing risk for developing non-fatal secondary outcomes and generate unadjusted hazard ratios (HR). To adjust for other covariates (pre-existing comorbidities, sex, race, and ethnicity), the Cox proportional hazards model was utilized, and adjusted hazard ratios (aHR) and their associated 95% confidence intervals (CI) were reported.

## 3. Results

**Figure 1:**
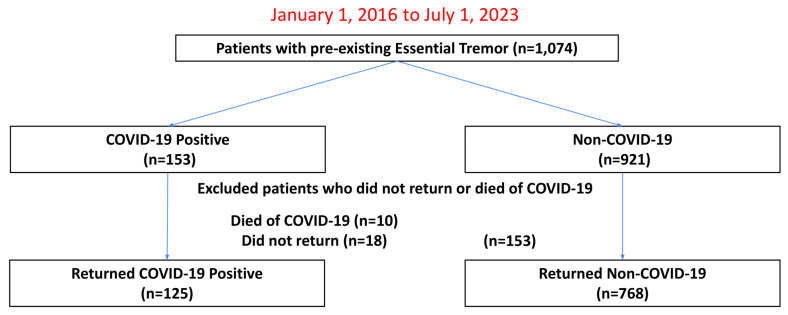
Patient selection flowchart.

Figure 1 shows the patient selection flowchart. Of the 1074 patients with pre-existing ET from 1 January 2016 to 1 July 2023, 153 had documented positive COVID-19 PCR tests, 10 of whom died of COVID-19, 18 of whom did not return to our health system, resulting in 125 COVID-19 patients who were followed for up to three and a half years after the start of the pandemic. There were 921 ET patients with no recorded history of positive COVID-19 PCR tests, of whom 153 did not return to our health system, resulting in 768 non-COVID ET controls.

Table 1 shows the demographics and clinical characteristics of patients with pre-existing ET included in our analysis. ET patients with documented COVID-19 infection did not differ from those without documented infection in terms of sex, age, ethnicity, and race. The COVID-19 group had a higher prevalence of type-2 diabetes, depression, and anxiety (*p* < 0.05 for all). There were not group differences for the remaining comorbidities. For COVID-19 patients with ET, 65.6% were hospitalized for COVID-19, 3 (2.4%) were treated for critical illness, 7.2% were treated with steroids, and 16.8% were treated with antiviral drugs.

**Figure 2:**
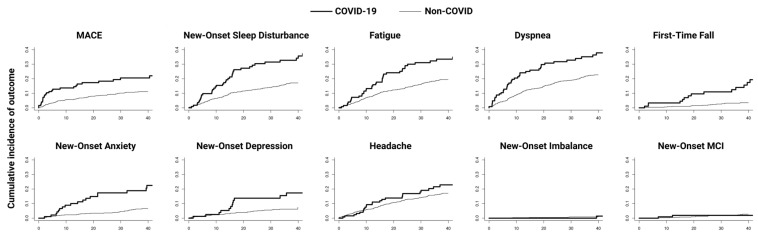
Cumulative incidence curves of post-index date outcomes of COVID-19 and non-COVID patients. MACE, major adverse cardiovascular event.

Cumulative incidence of post-index date MACE, sleep disturbances, fatigue, dyspnea, depression, anxiety, headache, and altered mental status and their univariate and multivariate Fine–Gray hazard ratios were computed (Figure 2). Multivariate analysis showed COVID-19 to be associated with MACEs (aHR = 2.39 [1.49, 3.82]), new-onset sleep disturbance (aHR = 2.12 [1.44, 3.13]), fatigue (aHR = 1.83 [1.27, 2.65]), dyspnea (aHR = 1.98 [1.40, 2.80]), first-time fall (aHR = 4.76 [2.24, 10.14]), new-onset anxiety (aHR = 3.66 [2.02, 6.64]), and new-onset depression (aHR = 2.38 [1.20, 4.70]) (Table 2).

**Figure 3:**
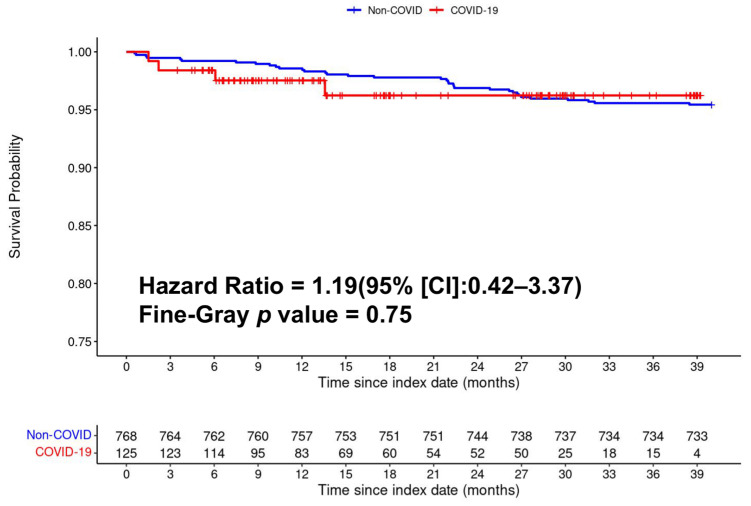
Kaplan–Meier curve of all-cause mortality fourteen days or more post-index date between COVID-19 and non-COVID patients.

All-cause mortality was not significantly different between the COVID-exposed and unexposed groups on the Kaplan–Meier survival curve (Figure 3). There were no significant group differences in all-cause mortality up to three and a half years post-index date (univariate HR = 1.19 [CI: 0.42, 3.37] *p* = 0.75) and multivariate HR = 0.64, 95%CI [0.19, 2.14], *p* < 0.47). Supplemental Table S2 shows the Cox proportional hazard ratios for all-cause mortality for other covariates.

## 4. Discussion

This analysis evaluated outcomes of patients with pre-existing ET up to three and a half years post-infection in a diverse urban population in the Bronx, New York. COVID-19 was linked with higher adjusted risk for MACEs, sleep disturbances, fatigue, dyspnea, first-time fall, depression, and anxiety in ET patients.

About 14% of ET patients had a positive PCR SARS-CoV-2 test, compared to an infection rate of 11% in MS [28], 12% in PD [47], and 21% in neurologically impaired [48] patients in our health system. Infection rates can vary by region, observation time of the study, the population, and testing rate. The Bronx was severely affected by early and subsequent waves of infection, and the underserved populations in the Bronx could be at higher risk of infection and worse outcomes compared to national averages [49]. ET patients with COVID-19 had similar demographic profiles and similar prevalence of comorbidities compared to COVID-unexposed patients, except that the COVID-19 group had higher prevalence of T2D, anxiety, and depression. Notably, 65.6% of patients with ET were hospitalized due to COVID-19, which is not surprising given the age of this cohort (66.57 ± 15.52 years). However, only 2.4% had COVID-19-related critical illness.

Increased risk of MACEs among COVID-19 patients has been reported in the general population [45,46,50], in those with PD [47], and in those with neurological disorders [48]. Some studies have reported cardiac involvement in ET, suggesting that ET and MACEs possibly share common risk factors such as chronic stress [51,52]. Therefore, it may not be surprising that in patients with ET, the acute inflammatory [53] and cardiac [46,50,54,55] effects of COVID-19 exacerbate the effects of these risk factors. After adjusting for some of these risk factors like CAD, CHF, and HTN, multivariate analysis still showed COVID-19 to be associated with higher risk (aHR = 2.14 [1.36, 3.38]) of MACEs in ET patients.

We also found that SARS-CoV-2 infection was linked with higher adjusted risk for sleep disturbances, fatigue, dyspnea, first-time fall, depression, and anxiety in ET patients. Although fatigue and dyspnea are symptoms that are non-specific to ET, several other symptoms have elevated prevalence in ET patients compared to age-matched controls [56], like sleep disturbances [57], falls [58], and psychiatric disorders [59]. Increased risk of depression and anxiety in the general COVID-19 population has also been reported previously [60,61,62] but not in the ET COVID-19 population. One study reported that those with COVID-19 had higher odds of developing severe anxiety (2.41, CI 2.01–2.90) and depression (3.64, CI 3.06–4.32) compared with non-COVID individuals at up to 8 months follow-up [63]. Another study found that the risk of a first psychiatric diagnosis was high 14 to 90 days after SARS-CoV-2. In our ET cohort, those with COVID-19 had a higher risk of first-time falls, which has also been reported in the general COVID-19 population [64] and is common in patients with ET [65]. It is not surprising that COVID-19 patients in our cohort had a higher risk of developing fatigue compared to non-COVID-exposed controls as this has been shown extensively in the general COVID-19 population [66]. In our ET cohort, COVID-19 increased the risk of developing sleep disturbances, an outcome often seen in those with long COVID [67]. Approximately half of the patients in our cohort were on a first- or second-line ET medication, which may have affected outcomes. However, the distribution was similar across COVID-19 and non-COVID groups.

SARS-CoV-2 infection did not increase post-infection mortality risk in ET patients. In contrast, COVID-19 has been previously associated with increased risk of all-cause mortality in survivors with MS [28], PD [47], and other common neurological conditions [48] in our health system. Given the profile of the PD [47] and neurological conditions [48] cohorts, we speculate this difference in findings may be in part due to the younger age and lower prevalence of comorbidities in the ET cohort, making ET patients relatively less susceptible to mortality. While the MS cohort [28] was considerably younger on average, the immune-mediated activity of MS may partially explain why MS patients may be more susceptible to long-term mortality post-COVID-19 as compared to ET patients.

Previous investigations found that in those with MS, SARS-CoV-2 is linked with higher long-term risk of all-cause mortality, optic neuritis [28], accelerated increase in scores on the disability scale [25], worsening of pre-existing neurological symptoms [27], new gadolinium-enhancing lesions on magnetic resonance imaging [25], and other unfavorable clinical outcomes [18,19,20,21,22,23,24,25,26]. In those with PD, SARS-CoV-2 survivors exhibit higher risk of new or worsened motor symptoms, dysphagia, cognition, fatigue, and sleep issues two months after the start of the pandemic [68]. Among PD patients, worsened motor function (as measured by Unified Parkinson’s Disease Rating Scale Part III) and increased levodopa equivalent daily dose have been reported at one-and-a-half-years post-infection [69]. Our data further support the notion that SARS-CoV-2 may accelerate neurodegenerative disease progression in patients with pre-existing neurological conditions.

### Limitations

There are several limitations. Findings in this study are limited to patients who returned to the health system after index date for routine checkups and office visits. The cohort studied had to test positive for SARS-CoV-2 in our health system, which may be biased to select for a higher proportion of more severe COVID-19 cases. Our population consisted of a low proportion of Whites and our findings might not be generalizable to populations that are less diverse. We did not analyze socioeconomic variables such as education and work status as they were not readily available. We did not analyze outcomes with respect to SARS-CoV-2 vaccinations, strain of SARS-CoV-2 and time of infection, and individual aspects of disease severity. Vaccination status could not be reliably obtained because patients could have been vaccinated outside of our health system. Reliance on medical records means mis-documentation or misdiagnosis was possible in both COVID-19 and non-COVID groups, and important covariates, some of which include ET severity, age of ET onset, results of motor tests, and ET treatments, were not consistently available and therefore not included. We performed power calculations and found that our sample sizes provided adequate statistical power to detect differences in all outcomes (power > 0.80 at α = 0.05) except for risks of all-cause mortality, MCI, and imbalance, for which the study was underpowered. More robust time-dependent modeling may improve risk estimates. As this is an observational study, subgroup analyses risk unreliable estimates and false positives. Due to the retrospective nature of this study, there could be residual confounding and other selection biases. Future studies should correlate COVID-19 hyperinflammatory markers with ET disease activity, track ET symptoms and progression prospectively post-COVID-19, as well as other clinical disorders [70,71,72,73,74].

## 5. Conclusions

This is the first cohort study that reported the long-term outcomes of ET patients with COVID-19 up to three and a half years post-infection. COVID-19 exposure significantly increases the risk of MACEs, sleep disturbances, fatigue, dyspnea, first-time fall, depression, and anxiety in patients with pre-existing ET. Knowledge of the potential long-term negative impacts on patients with ET and identification of risk factors could bring awareness to enable better management of long COVID-19 sequelae among patients with ET.

## Figures and Tables

**Table 1:** Demographics and baseline characteristics of essential tremor patients.

	Patients with Pre-Existing Essential Tremor (N = 893)		*p* Values
	COVID-19 (N = 125)	Non-COVID-19 (N = 768)	
Age at Index Date, mean ± SD (years)	66.57 ± 15.52	68.15 ± 17.66	0.30
Female, n (%)	72 (57.60)	448 (58.33)	0.96
**Race and Ethnicity, n (%)**			
White	30 (24.00)	170 (22.14)	0.73
Black	36 (28.80)	216 (28.13)	0.96
Asian	4 (3.20)	26 (3.39)	1.00
Other Races	55 (44.00)	356 (46.35)	0.69
Hispanic	53 (42.40)	297 (38.67)	0.49
**Pre-Existing Conditions, n (%)**			
Hypertension	94 (75.20)	558 (72.66)	0.63
Type-2 diabetes	79 (63.20)	395 (51.43)	0.019
Chronic obstructive pulmonary disease	17 (13.60)	84 (10.94)	0.47
Asthma	27 (21.60)	154 (20.05)	0.78
Congestive heart failure	16 (12.80)	116 (15.10)	0.59
Coronary artery disease	26 (20.80)	148 (19.27)	0.78
Chronic kidney disease	44 (35.20)	203 (26.43)	0.054
Tobacco use	57 (45.60)	334 (43.49)	0.73
Obesity	47 (37.60)	246 (32.03)	0.26
Sleep disturbances	12 (9.60)	96 (12.50)	0.44
Depression	45 (36.00)	88 (11.46)	<0.005
Anxiety	33 (26.40)	92 (11.98)	<0.005
Headache	17 (13.60)	90 (11.72)	0.65
History of falls	35 (28.00)	239 (31.12)	0.55
Imbalance	3 (2.40)	19 (2.47)	1.00
Mild cognitive impairment or dementia	15 (12.00)	106 (13.80)	0.69
**ET Medications, n (%)**			
First Line	56 (44.80)	402 (52.34)	0.14
Second Line	75 (60.00)	391 (50.91)	0.073
**Acute COVID-19 Treatments, n (%)**			
Hospitalized	82 (65.60)	-	-
Critical illness	3 (2.40)	-	-
Steroids	21 (7.20)	-	-
Antiviral drugs	9 (16.80)	-	-

**Table 2:** Fine–Gray univariate and multivariate hazard ratios of COVID-19 and non-COVID patients. MACE, major adverse cardiovascular event.

Outcomes	Univariate			Multivariate		
	COVID-19 HR	95% [CI]	*p* Value	COVID-19 aHR	95% [CI]	*p* Value
MACEs	2.14	1.36–3.38	<0.005	2.39	1.49–3.82	<0.005
New-onset sleep disturbance	2.29	1.57–3.35	<0.005	2.12	1.44–3.13	<0.005
Fatigue	1.97	1.37–2.82	<0.005	1.83	1.27–2.65	<0.005
Dyspnea	1.94	1.38–2.73	<0.005	1.98	1.40–2.80	<0.005
First-time fall	5.10	2.52–10.31	<0.005	4.76	2.24–10.14	<0.005
New-onset anxiety	3.74	2.13–6.56	<0.005	3.66	2.02–6.64	<0.005
New-onset depression	2.74	1.41–5.34	<0.005	2.38	1.20–4.70	0.013
Headache	1.42	0.93–2.18	0.10	1.42	0.91–2.21	0.12
New-onset imbalance	1.05	0.14–7.93	0.96	1.13	0.12–10.34	0.91
New-onset MCI	0.86	0.21–3.55	0.84	0.85	0.19–3.75	0.83

## Data Availability

The original contributions presented in this study are included in the article/Supplementary Materials. Further inquiries can be directed to the corresponding author.

## Supplementary Materials

The following supporting information can be downloaded at: https://www.mdpi.com/article/10.3390/diagnostics14242774/s1, Table S1: OMOP Concept Names and Concept IDs Used; Table S2: Cox proportional hazard ratios (HR) for post-infection all-cause mortality.
