# Supplementary material for: Long-Term Outcomes of Patients with Pre-Existing Essential Tremor After SARS-CoV-2 Infection
Source: Diagnostics (Basel). 2024 Dec 10;14(24):2774. doi: 10.3390/diagnostics14242774 (PMC11674104; doi:10.3390/diagnostics14242774)
Supplement: Supplementary file 1 [file diagnostics-14-02774-s001.zip › diagnostics-3313521-supplementary.pdf]

**Supplementary Table 1.** OMOP Concept Names and Concept IDs Used

| OMOP Concept Name | OMOP Concept ID |
|---|---|
| **COVID-19 Test** | |
| SARS-CoV-2 (COVID-19) RNA [Presence] in Respiratory specimen by NAA with probe detection | 706163 |
| SARS-CoV-2 (COVID-19) RNA [Presence] in Unspecified specimen by NAA with probe detection | 706170 |
| **Positive Result of COVID-19 Test** | |
| Positive | 9191 |
| Detected | 45877985 |
| Positive | 45884084 |
| **Essential Tremor** | |
| Essential tremor | 43531003 |
| **Hypertension** | |
| Pre-eclampsia or eclampsia with pre-existing hypertension | 141084 |
| Portal hypertension | 192680 |
| Benign intracranial hypertension | 312902 |
| Renovascular hypertension | 317895 |
| Secondary hypertension | 319826 |
| Essential hypertension | 320128 |
| Pre-existing hypertension complicating pregnancy, childbirth and puerperium | 321074 |
| Renal hypertension | 443771 |
| Pulmonary arterial hypertension | 4013643 |
| Pre-existing secondary hypertension complicating pregnancy, childbirth and puerperium | 4057979 |
| Neonatal hypertension | 4071202 |
| Hypertension secondary to endocrine disorder | 4110948 |
| Maternal hypertension | 4118910 |
| Chronic thromboembolic pulmonary hypertension | 4120094 |
| Persistent pulmonary hypertension of the newborn | 4121462 |
| Pregnancy-induced hypertension | 4167493 |
| Pre-existing hypertension in obstetric context | 4311246 |
| Chronic peripheral venous hypertension | 4313767 |
| Pulmonary hypertension | 4322024 |
| Secondary pulmonary hypertension | 4339214 |
| Hypertension complicating pregnancy | 42538946 |

| | |
|---|---|
| Pulmonary hypertension due to left heart disease | 43020910 |
| Chronic kidney disease due to hypertension | 44782429 |
| Chronic peripheral venous hypertension with lower extremity complication | 44782715 |
| Pulmonary hypertension due to lung disease and/or hypoxia | 44783628 |
| Postpartum pregnancy-induced hypertension | 45757788 |
| **Type-2 Diabetes** | |
| Hyperosmolar coma due to type 2 diabetes mellitus | 201530 |
| Type 2 diabetes mellitus | 201826 |
| Disorder of nervous system due to type 2 diabetes mellitus | 376065 |
| Peripheral circulatory disorder due to type 2 diabetes mellitus | 443729 |
| Renal disorder due to type 2 diabetes mellitus | 443731 |
| Disorder of eye due to type 2 diabetes mellitus | 443733 |
| Pre-existing type 2 diabetes mellitus | 4063043 |
| Type 2 diabetes mellitus with ulcer | 4099651 |
| Lumbosacral radiculoplexus neuropathy due to type 2 diabetes mellitus | 4140466 |
| Type 2 diabetes mellitus without complication | 4193704 |
| Arthropathy due to type 2 diabetes mellitus | 4196141 |
| Cataract due to diabetes mellitus type 2 | 4221495 |
| Mononeuropathy due to type 2 diabetes mellitus | 4222415 |
| Gangrene due to type 2 diabetes mellitus | 4222876 |
| Ketoacidotic coma due to type 2 diabetes mellitus | 4228443 |
| Hypoglycemic coma due to type 2 diabetes mellitus | 36714116 |
| Hyperglycemia due to type 2 diabetes mellitus | 37016349 |
| Autonomic neuropathy due to type 2 diabetes mellitus | 37016768 |
| Polyneuropathy due to type 2 diabetes mellitus | 37017432 |
| Nonproliferative retinopathy due to type 2 diabetes mellitus | 43530656 |
| Proliferative retinopathy due to type 2 diabetes mellitus | 43530685 |
| Foot ulcer due to type 2 diabetes mellitus | 43530690 |
| Pre-existing type 2 diabetes mellitus in pregnancy | 43531010 |
| Neuropathic arthropathy due to type 2 diabetes mellitus | 43531563 |
| Chronic kidney disease due to type 2 diabetes mellitus | 43531578 |
| Dermopathy due to type 2 diabetes mellitus | 43531616 |
| Hypoglycemia due to type 2 diabetes mellitus | 45757363 |

| | |
|---|---|
| Mild nonproliferative retinopathy due to type 2 diabetes mellitus | 45757435 |
| Macular edema and retinopathy due to type 2 diabetes mellitus | 45770830 |
| Moderate nonproliferative retinopathy due to type 2 diabetes mellitus | 45770881 |
| Traction detachment of retina due to type 2 diabetes mellitus | 45773064 |
| **Chronic Obstructive Pulmonary Disease** | |
| Acute exacerbation of chronic obstructive airways disease | 257004 |
| Chronic obstructive pulmonary disease with acute lower respiratory infection | 4110056 |
| **Asthma** | |
| Cough variant asthma | 313236 |
| Asthma | 317009 |
| Exacerbation of intermittent asthma | 4138760 |
| Mild intermittent asthma | 4146581 |
| Eosinophilic asthma | 4279553 |
| Acute severe refractory exacerbation of asthma | 37116845 |
| Uncomplicated asthma | 45768910 |
| Uncomplicated mild persistent asthma | 45768963 |
| Uncomplicated moderate persistent asthma | 45768964 |
| Uncomplicated severe persistent asthma | 45768965 |
| Acute severe exacerbation of severe persistent asthma | 45769350 |
| Acute severe exacerbation of moderate persistent asthma | 45769351 |
| Acute severe exacerbation of mild persistent asthma | 45769352 |
| Acute severe exacerbation of asthma | 45769438 |
| Acute exacerbation of asthma | 45771045 |
| Acute exacerbation of mild persistent asthma | 46270082 |
| Acute exacerbation of moderate persistent asthma | 46273487 |
| **Cardiovascular Disease** | |
| Heart failure | 316139 |
| Hypertensive heart disease without congestive heart failure | 319034 |
| Congestive heart failure | 319835 |
| Hypertensive heart and renal disease with (congestive) heart failure | 439696 |
| Left heart failure | 439846 |
| Systolic heart failure | 443580 |
| Diastolic heart failure | 443587 |

| | |
|---|---|
| Hypertensive heart failure | 444101 |
| High output heart failure | 4004279 |
| Chronic right-sided heart failure | 4014159 |
| Right heart failure secondary to left heart failure | 4195785 |
| Chronic congestive heart failure | 4229440 |
| Acute right-sided heart failure | 4233424 |
| Biventricular congestive heart failure | 4242669 |
| Acute on chronic right-sided congestive heart failure | 37309625 |
| Chronic systolic heart failure | 40479192 |
| Chronic diastolic heart failure | 40479576 |
| Acute on chronic systolic heart failure | 40480602 |
| Acute systolic heart failure | 40480603 |
| Acute diastolic heart failure | 40481042 |
| Acute on chronic diastolic heart failure | 40481043 |
| Acute combined systolic and diastolic heart failure | 44782718 |
| Chronic combined systolic and diastolic heart failure | 44782719 |
| Acute on chronic combined systolic and diastolic heart failure | 44782733 |
| Arteriosclerosis of coronary artery bypass graft | 443563 |
| Atherosclerosis of coronary artery without angina pectoris | 764123 |
| Chronic total occlusion of coronary artery | 36712779 |
| Dissection of coronary artery | 37115756 |
| Arteriosclerosis of coronary artery bypass graft of transplanted heart | 40481132 |
| Arteriosclerosis of autologous vein coronary artery bypass graft | 40482638 |
| Arteriosclerosis of nonautologous coronary artery bypass graft | 40482655 |
| Coronary artery graft present | 42537730 |
| Mechanical breakdown of coronary artery bypass graft | 43020458 |
| Acute ST segment elevation myocardial infarction involving left anterior descending coronary artery | 43020460 |
| Arteriosclerosis of autologous arterial coronary artery bypass graft | 43021857 |
| Acute ST segment elevation myocardial infarction due to left coronary artery occlusion | 46270162 |
| Acute ST segment elevation myocardial infarction due to right coronary artery occlusion | 46270163 |
| **Tobacco Use** | |
| Tobacco use and exposure | 4041306 |
| **Obesity** | |

| | |
|---|---|
| Obesity | 433736 |
| Morbid obesity | 434005 |
| Maternal obesity syndrome | 439893 |
| Drug-induced obesity | 4097996 |
| Extreme obesity with alveolar hypoventilation | 4100857 |
| Maternal obesity complicating pregnancy, childbirth and the puerperium, antepartum | 42872398 |
| Obesity in mother complicating childbirth | 45757112 |
| **Major Adverse Cardiovascular Event** | |
| Subarachnoid hemorrhage | 432923 |
| Atrial septal defect due to and following acute myocardial infarction | 438172 |
| Cerebral infarction | 443454 |
| Subcortical hemorrhage | 4049659 |
| Rupture of chordae tendinae due to and following acute myocardial infarction | 4108219 |
| Rupture of papillary muscle as current complication following acute myocardial infarction | 4108220 |
| Cerebral infarction due to embolism of cerebral arteries | 4108356 |
| Hemopericardium due to and following acute myocardial infarction | 4108678 |
| Rupture of cardiac wall without hemopericardium as current complication following acute myocardial infarction | 4108679 |
| Thrombosis of atrium, auricular appendage, and ventricle due to and following acute myocardial infarction | 4108680 |
| Subarachnoid hemorrhage from carotid siphon and bifurcation | 4108952 |
| Intracerebral hemorrhage, multiple localized | 4110186 |
| Cerebral infarct due to thrombosis of precerebral arteries | 4110189 |
| Cerebral infarction due to embolism of precerebral arteries | 4110190 |
| Cerebral infarction due to thrombosis of cerebral arteries | 4110192 |
| Subarachnoid hemorrhage from vertebral artery | 4111708 |
| Non-traumatic subdural hemorrhage | 4111709 |
| Cerebral infarction due to cerebral venous thrombosis, non-pyogenic | 4111714 |
| Non-traumatic intracerebral ventricular hemorrhage | 4144154 |
| Spontaneous subarachnoid hemorrhage | 4148906 |
| Cortical hemorrhage | 4176892 |
| Acute non-ST segment elevation myocardial infarction | 4270024 |
| Acute ST segment elevation myocardial infarction | 4296653 |
| Brain stem hemorrhage | 4319328 |

| | |
|---|---|
| Acute on chronic right-sided congestive heart failure | 37309625 |
| Myocardial infarction due to demand ischemia | 37309626 |
| Acute on chronic systolic heart failure | 40480602 |
| Acute on chronic diastolic heart failure | 40481043 |
| Spontaneous hemorrhage of cerebral hemisphere | 42535425 |
| Acute ST segment elevation myocardial infarction involving left anterior descending coronary artery | 43020460 |
| Spontaneous cerebellar hemorrhage | 43530674 |
| Spontaneous cerebral hemorrhage | 43530727 |
| Acute on chronic combined systolic and diastolic heart failure | 44782733 |
| Cerebral infarction due to thrombosis of middle cerebral artery | 45767658 |
| Cerebral infarction due to embolism of middle cerebral artery | 45772786 |
| Cerebral infarction due to occlusion of precerebral artery | 46270031 |
| Acute ST segment elevation myocardial infarction due to left coronary artery occlusion | 46270162 |
| Acute ST segment elevation myocardial infarction due to right coronary artery occlusion | 46270163 |
| Cerebral infarction due to occlusion of basilar artery | 46273649 |
| **Sleep Disturbances** | |
| Sleep-wake schedule disorder, delayed phase type | 433467 |
| Sleep disorder | 435524 |
| Disorder of sleep-wake cycle | 435786 |
| Irregular sleep-wake pattern | 436522 |
| Insomnia | 436962 |
| REM sleep behavior disorder | 439007 |
| Central sleep apnea syndrome | 439794 |
| Non-24 hour sleep-wake cycle | 440092 |
| Sleep-wake schedule disorder, advanced phase type | 440384 |
| Not getting enough sleep | 4087475 |
| Nonorganic insomnia | 4102985 |
| Psychophysiologic insomnia | 4138617 |
| Sleep state misperception | 4175644 |
| Primary insomnia | 4215402 |
| Sleep terror disorder | 4232324 |
| Idiopathic hypersomnia without long sleep time | 40482713 |
| Idiopathic hypersomnia associated with long sleep time | 40483183 |

| | |
|---|---|
| Behavioral insomnia of childhood, limit setting type | 43020464 |
| Behavioral insomnia of childhood, sleep onset association type | 43020467 |
| Behavioral insomnia of childhood | 43021812 |
| Primary central sleep apnea | 43022069 |
| Daytime dozing / sleeping (narcolepsy) | 35810207 |
| Trouble falling or staying asleep, or sleeping too much | 35811202 |
| Trouble falling or staying asleep, or sleeping too much in last 2 weeks.presence [Reported PHQ-9 CMS] | 40757771 |
| I didn't sleep as well as I usually sleep [CES-DC] | 40768167 |
| Trouble falling or staying asleep in the last month [PCL-C] | 42868707 |
| Sleep disturbances indicator in the past week [UPDRS] | 46236402 |
| **Fatigue** | |
| Chronic fatigue syndrome | 432738 |
| Fatigue | 4223659 |
| Recent feelings of tiredness or low energy | 35811204 |
| Feeling tired or having little energy in last 2 weeks.frequency [Reported PHQ-9 CMS] | 40757774 |
| I felt like I was too tired to do things [CES-DC] | 40768163 |
| **Dyspnea** | |
| Dyspnea | 312437 |
| **Imbalance** | |
| Cerebellar ataxia | 435242 |
| Ataxia | 437584 |
| Incoordination | 441417 |
| Late onset cerebellar ataxia | 4041682 |
| Early onset cerebellar ataxia | 4047635 |
| Coordination problem | 4114624 |
| Cerebellar disorder | 4329160 |
| **Depression** | |
| Recurrent major depressive episodes, severe, with psychosis | 434911 |
| Severe recurrent major depression without psychotic features | 435220 |
| Severe major depression, single episode, with psychotic features | 438406 |
| Severe major depression, single episode, without psychotic features | 441534 |
| Moderate major depression, single episode | 4049623 |
| Moderate recurrent major depression | 4077577 |

| | |
|---|---|
| Recurrent depression | 4098302 |
| Mild major depression, single episode | 4195572 |
| Mild recurrent major depression | 4228802 |
| Major depression, single episode | 4282096 |
| Recurrent major depression | 4282316 |
| Depression screening using PHQ-9 (Patient Health Questionnaire 9) score | 37395717 |
| **Anxiety** | |
| Organic anxiety disorder | 381537 |
| Generalized anxiety disorder | 434613 |
| Anxiety disorder | 442077 |
| Generalized anxiety disorder 7 item score | 40483769 |
| **Headache** | |
| Migraine | 318736 |
| Refractory migraine with aura | 372886 |
| Cough headache syndrome | 373463 |
| Idiopathic stabbing headache | 374922 |
| Headache disorder | 375527 |
| Tension-type headache | 376382 |
| Episodic tension-type headache | 377545 |
| Chronic tension-type headache | 377853 |
| Chronic cluster headache | 378145 |
| Migraine without aura | 378735 |
| Episodic cluster headache | 380093 |
| Cluster headache | 381278 |
| Migraine with aura | 381549 |
| Hemiplegic migraine | 433763 |
| Refractory migraine | 443615 |
| Refractory migraine without aura | 443616 |
| Vascular headache | 4134454 |
| Primary thunderclap headache | 43530641 |
| New daily persistent headache | 43530648 |
| Chronic intractable migraine without aura | 43530652 |
| Migraine with persistent visual aura | 44782470 |

| | |
|---|---|
| Headaches [NDI] | 21493489 |
| **Fall** | |
| Fall from ladder | 432803 |
| Fall from furniture | 433657 |
| Fall from toilet seat | 434549 |
| Accidental fall | 435991 |
| Fall | 436583 |
| Fall from bed | 436882 |
| Fall on same level from slipping, tripping or stumbling | 437175 |
| Fall from playground equipment | 437477 |
| Fall on or from stairs or steps | 441749 |
| Falls | 4059015 |
| Fall on same level | 4140830 |
| Fall in bathtub | 4236512 |
| Fall from chair | 4308870 |
| **Mild Cognitive Impairment or Dementia** | |
| Minimal cognitive impairment | 439795 |
| Alzheimer's disease | 378419 |
| Primary degenerative dementia of the Alzheimer type, presenile onset | 4218017 |
| Primary degenerative dementia of the Alzheimer type, senile onset | 4220313 |
| Dementia associated with another disease | 374888 |
| Dementia associated with alcoholism | 378726 |
| Psychoactive substance-induced organic dementia | 4009647 |
| Dementia | 4182210 |
| Sedative, hypnotic AND/OR anxiolytic-induced persisting dementia | 4245766 |
| Dementia with behavioral disturbance | 43530666 |
| Vascular dementia with behavioral disturbance | 37018688 |
| Vascular dementia without behavioral disturbance | 37109056 |
| Frontotemporal dementia | 4043378 |
| Diffuse Lewy body disease | 380701 |

**Supplemental Table 2.** Cox proportional hazard ratios (HR) for post-infection all-cause mortality.

| Outcome | HR | 95% [CI] | *P* value |
|---|---|---|---|
| SARS-CoV-2 Infection | 0.64 | 0.19 – 2.14 | 0.47 |
| Age at Index Date | 1.04 | 1.00 – 1.07 | **0.03** |
| Female vs. Male | 1.35 | 0.65 – 2.83 | 0.42 |
| Black vs. White | 0.75 | 0.28 – 2.02 | 0.57 |
| Asian vs. White | 1.20 | 0.14 – 10.28 | 0.87 |
| Other Race vs. White | 1.20 | 0.45 – 3.19 | 0.71 |
| Hispanic vs. White | 1.14 | 0.47 – 2.79 | 0.77 |
| Hypertension | 4.87 | 0.63 – 37.4 | 0.13 |
| Type-2 Diabetes | 1.64 | 0.70 – 3.85 | 0.25 |
| CHF | 3.29 | 1.57 – 6.90 | **<0.005** |
| COPD | 1.26 | 0.51 – 3.07 | 0.62 |
| Asthma | 0.57 | 0.23 – 1.40 | 0.22 |
| Coronary Artery | 1.46 | 0.71 – 3.02 | 0.30 |
| Chronic Kidney Disease | 0.91 | 0.45 – 1.82 | 0.79 |
| Tobacco Use | 1.23 | 0.61 – 2.49 | 0.57 |
| Obesity | 1.46 | 0.72 – 2.99 | 0.29 |
